# Optimization and Production of Aceclofenac-Loaded Microfiber Solid Dispersion by Centrifugal Spinning

**DOI:** 10.3390/pharmaceutics15092256

**Published:** 2023-08-31

**Authors:** Enikő Bitay, Attila Levente Gergely, Zoltán-István Szabó

**Affiliations:** 1 Department of Mechanical Engineering, Faculty of Technical and Human Sciences, Sapientia Hungarian University of Transylvania, Calea Sighișoarei nr. 2., 540485 Târgu-Mureş, Romania; bitay.eniko@eme.ro; 2 Research Institute of the Transylvanian Museum Society, 2–4 Napoca, 400009 Cluj, Romania; 3 Department of Drugs Industry and Pharmaceutical Management, George Emil Palade University of Medicine, Pharmacy, Science, and Technology of Targu Mures, Gh. Marinescu 38, 540485 Târgu-Mureş, Romania; zoltan.szabo@umfst.ro; 4 Sz-imfidum Ltd., 525401 Lunga, Romania

**Keywords:** aceclofenac, solid dispersion, fibers, centrifugal spinning, drug delivery system

## Abstract

Aceclofenac-loaded polyvinylpyrrolidone fiber-based amorphous solid dispersion was produced successfully by centrifugal spinning. The solution concentration and rotational speed were optimized to produce the fiber-based drug carrier system, with a determined production rate of 12.7 g/h dry solid fibers. The obtained fibers were bead-free and smooth-surfaced with an average diameter of 7.5 ± 2.5 μm. Gas chromatographic determinations revealed that ethanol, as a residual solvent, was well below the regulatory limit of 0.5%. Differential scanning calorimetric investigation and infrared spectroscopic measurements were used to track the physicochemical changes that intervene during fiber formation in the solid state. The results revealed that the rapid evaporation of the solvent was accompanied by a probable crystalline to amorphous transition of the active substance during centrifugal spinning. In vitro dissolution studies revealed an instantaneous disintegration of the fibrous structure and a rapid release of the active substance, with the microfibrous webs greatly outperforming the crystalline active substance, especially in the early time-points. This implies that centrifugal spinning offers a viable scale-up production process to prepare drug-loaded fiber-based solid dispersions.

## 1. Introduction

Polymer nanofibers with fiber diameters of a couple of hundred nanometers have received much attention in the past couple of decades due to their unique properties resulting from their small size. Such properties as flexibility, porosity, and a high surface-to-volume ratio inherently make these types of structures very promising in several fields, such as energy generation and storage, filtration (separation), sensor technology, carriers for catalysts, and biomedical applications [1,2]. In particular, in biomedical applications such as wound dressing, scaffolds, and drug delivery systems, the nano- and microfiber-based structures are very promising and innovate the field producing not only countless publications but also patents.

Electrospinning is the most popular technique to produce nanofibers due to its ease of use and relatively low cost [3]. The fiber production process via electrospinning has been described earlier in detail [4,5]. Briefly, the electrospinning process uses an electric field to produce fibers. During the process, a cone-like shaped droplet forms at the tip of a capillary (due to the used electric field), termed Taylor cone [6]. When the tension produced by the repulsive forces surpasses the surface tension of the polymer solution, a jet forms. This jet will travel towards a grounded collector due to the electric field. Most of the used solvent evaporates during the process; thus, dry fibers can deposit and accumulate on the collector, creating a non-woven porous fiber mat [7,8]. The electrospinning fiber production process has the advantage of using a simple and flexible setup, fibers with nanometer-scale diameters can be acquired relatively easily, the fiber diameter can be controlled, and a large literature body is available. However, there are several major disadvantages of this technique, such as the use of a relatively high voltage, up to 50 kV, the used solution has to be electrically conductive, and perhaps the most significant of all is the very low production rate, 0.01–0.1 g/h dry fiber, using the single needle setup [9,10].

Several methods have been developed to improve the fiber production rate of electrospinning. All of the methods increase the number of Taylor cones during the process. These techniques could be divided into two major categories: multi-needle techniques and needle-free electrospinning [11]. The former involves the use of multiple needles that inherently increase the complexity of the operational and cleaning processes [12,13], whereas the latter uses a large surface of polymer solution [14]. Different methods and spinnerets have been designed that could be used to create multiple Taylor cones on the surface of the polymer solution [15,16,17,18,19,20,21,22]. However, these processes all have the disadvantage of using a large free surface of polymer solution, and thus water absorption and/or solvent evaporation becomes a pressing issue. Both phenomena change the composition of the used polymer solution, and thus the fiber production process is influenced uncontrollably. Furthermore, needleless electrospinning requires the use of high voltage for the fiber production process, generally >50 kV [21]. The electric conductivity of the solution could also be a limiting factor. For fiber-based drug delivery systems, a balance has to be maintained between the drug and carrier solubility and the electrical conductivity of the prepared polymer–drug–solvent system, which could be challenging in some cases. To facilitate the required electrical conductivity for non-conductive polymer/solvent systems, additives have been used [23]. However, the use of additives makes the solution system more complex, and for biomedical applications, the additive’s toxicity should be considered.

**Figure 1:**
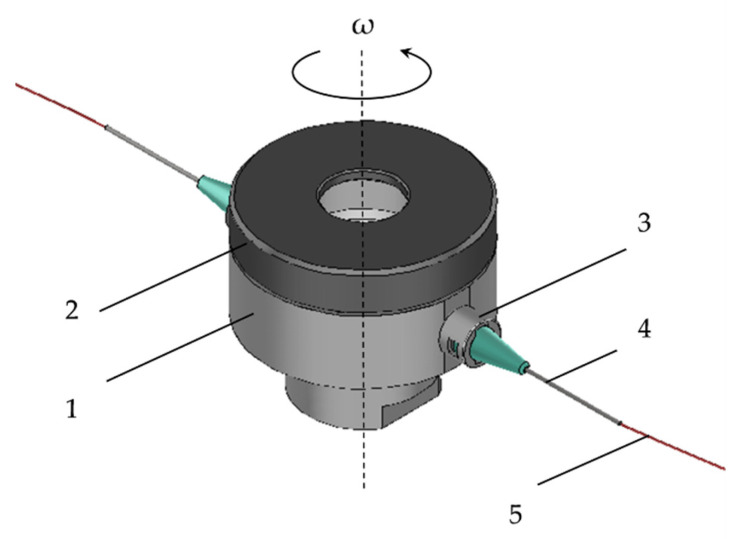
Custom-designed centrifugal spinning rotating head: 1—lower part, 2—cap, 3—thread-to-lure connector, 4—needle, and 5—polymer jet.

An alternative fiber production method to electrospinning could be the so-called centrifugal spinning (or Forcespinning™, rotary jet-spinning). Centrifugal spinning uses the centrifugal force to generate fibers [24]. The working principle of centrifugal spinning involves the use of a rotating chamber (or head) that contains the polymer solution and has capillaries placed radially, as shown in Figure 1.

During the fiber production process, the rotating head rotates at a speed in the range of 3000–15,000 rpm. The polymer solution exits from the rotating chamber through the capillary and a jet forms. The jet is elongated by the centrifugal and partially by the Coriolis forces that are present due to the rotating motion. While the jet travels towards the collector (vertical roads placed radially around the rotating head at a certain distance), the solvent evaporates, resulting in the deposition of dry polymer fibers on the collector. The literature data show that in principle, there is little difference in fiber diameters when electrospinning and centrifugal spinning are compared head-to-head under optimized laboratory conditions [25]. Most importantly, it has been shown that the production rate of centrifugal spinning at a laboratory scale could be as high as 60 g/h per capillary, which is two to three orders of magnitude larger than in the case of the single needle electrospinning [26,27]. Furthermore, the electric conductivity of the used solution does not influence the fiber production process [28].

**Figure 2:**
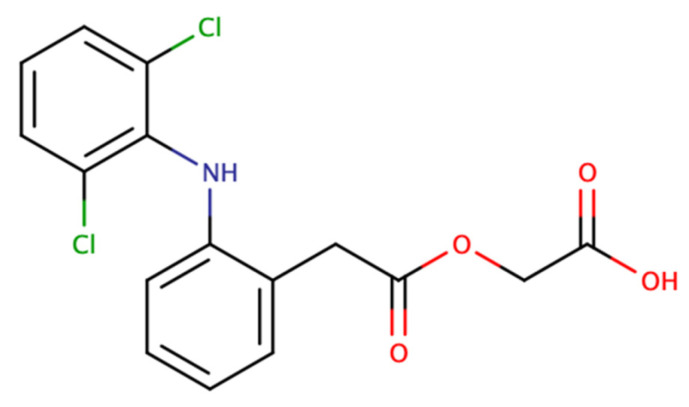
Chemical structure of aceclofenac.

In the present study, we have selected aceclofenac as a model drug to develop an aceclofenac-loaded fiber-based drug delivery system with centrifugal spinning. Aceclofenac (Figure 2) is an orally active, non-steroidal, anti-inflammatory drug (NSAID). It acts as a cyclooxygenase (COX) enzyme inhibitor, with a preference for the COX-2 isoform, thus presenting potent anti-inflammatory and analgesic properties, with better gastrointestinal tolerability than diclofenac [29,30,31]. It is used for the management of moderate-to-severe pain and in different arthritic disorders. According to the Biopharmaceutical Classification System (BCS), aceclofenac is classified as a Class II drug, with low solubility and high permeability. It is characterized by low bioavailability because of its low solubility and extensive metabolism [32].

The bioavailability and solubility of BSC Class II drugs can be increased by size reduction, the use of water-soluble complexes, the application of surfactants, or the development of solid dispersions [33]. Polymer fiber-based solid dispersions gained a substantial focus in the literature partially due to the high porosity and surface-to-volume ratio properties. It has been shown that the drug is in its amorphous state in the polymer fiber-based solid dispersions [34].

The oral bioavailability of aceclofenac has been improved by the development of chitosan alginate cocrystals [35], the use of polymeric microspheres for sustained drug release [36], the use of nanocrystals prepared via the surface solid dispersion method [37], or the development of interpenetrating network nanocomposites [38]. Also, aceclofenac-loaded polyvinyl pyrrolidone (PVP) fiber-based solid dispersions using the electrospinning process have been developed [39]. Based on the results, the produced fibers had an average fiber diameter of d = 596 ± 215 nm at a production rate of 0.07 g/h dry fiber. In order to be able to use the electrospinning process, low aceclofenac dosage could be used, only 5.88 wt% with respect to the amount of carrier, PVP. Furthermore, the addition of triethanolamine was needed to facilitate the electrospinning process. Based on the dissolution results, the aceclofenac dissolved much faster from the nanofiber-based delivery system in contrast to the pure drug.

The aim of this work is to investigate the production process of aceclofenac-loaded fiber-based solid dispersion using centrifugal spinning. Furthermore, the present study discusses the optimization of the fiber production method and examines the dissolution characteristics of the drug-loaded fibers.

## 2. Materials and Methods

### 2.1. Materials

Aceclofenac (Figure 2) was provided by Richter Gedeon Plc. (Budapest, Hungary), poly (vinyl-pyrrolidone) (PVP K90, Kollidon K90) was obtained from Sigma Aldrich (Merck, Darmstadt, Germany), and ethanol (EtOH, 96%) was from Chemical Company (Iasi, Romania). Methanol, acetonitrile, and orto-phosphoric acid (85%) used during the liquid chromatographic measurements were HPLC grade, obtained from Merck (Darmstadt, Germany). Ultrapure distilled water was prepared in-house by a MilliQ water purification system. Potassium dihydrogen phosphate and sodium hydroxide used throughout the in vitro dissolution studies were obtained from Merck (Darmstadt, Germany).

### 2.2. Polymer Solution Preparation

PVP solutions in ethanol at different concentrations were prepared at room temperature. The solution was stirred at 1000 rpm with a magnetic stirrer (ARE 1500, Fisher Scientific, Loughborough, UK) for 1 h to result in a transparent viscous solution.

Aceclofenac was dissolved in ethanol through magnetic stirring (ARE 1500, Fisher Scientific) for 15 min at room temperature resulting in a clear solution. The fiber-forming polymer, PVP K90, was added in small aliquots to the previously obtained solution under magnetic stirring at 1000 rpm at room temperature. After 1 h of stirring, a clear, viscous solution was obtained. The final concentration of the solution was 4.8 wt% aceclofenac and 23.8 wt% PVP in ethanol.

### 2.3. Centrigual Spinning

Centrifugal spinning was performed on an in-house built setup. The rotational movement was insured by an angle grinder (GWX13-50VSP, Bosch, Germany) that had the possibility to change the rotational speed between 1000–9000 rpm. A custom rotating head was designed based on the work of Sebe et al. [40]. The 3D model of the rotating head can be seen in Figure 1.

The custom-built rotating head consists of several parts: the lower part, nr. 1, in Figure 1, holds the polymer solution. This part is made of Al to prevent corrosion yet ensure durability and light weight. The cap (nr. 2), made of PA6, is necessary to keep the solution in the rotating head during the centrifugal spinning process. The rotating head is equipped with two thread-to-lure connectors (nr. 3), and thus the needles (nr. 4) could be changed to any size.

Centrifugal spinning was carried out using G24 needles and the needle-to-collector distance was set to 150 mm. All experiments were performed at room temperature (~21 °C) and at ~35% relative humidity.

### 2.4. Scanning Electron Microscopy (SEM)

SEM was performed on a JOEL JSM-5200 (JOEL, Tokyo, Japan) scanning electron microscope at 1 kV accelerating voltage. The samples were used as is, without sputter coating. The low accelerating voltage was necessary due to the loose structure of the fiber mat. The average fiber diameter (using the normal distribution equation) was determined using the ImageJ software (version 1.52a, National Institutes of Health, Bethesda, MD, USA) based on 100 random measurements on multiple SEM images that were taken from different parts of the sample.

### 2.5. Differential Scanning Calorimetry (DSC)

DSC measurements were performed using a Shimadzu DSC-60 (Shimadzu, Tokyo, Japan) calorimeter. Samples of 3–10 mg were placed into aluminum pans and the pans were sealed. The reference was an empty aluminum pan of the same type. The samples were subjected to heating in the temperature range of 25 to 200 °C with a heating rate of 2 °C/min.

### 2.6. Fourier Transform Infrared (FT-IR) Spectroscopy

FT-IR measurements were performed on a Bruker Tensor 27 IR spectrometer (Bruker Optics, Ettlingen, Germany), controlled by the Opus software (version 7.2). IR spectra of the individual components (aceclofenac, PVP K90), their physical mixture, and the microfibrous sample were collected in transmittance mode over the 400–4000 cm^−1^ wavenumber range. Each of the mentioned samples was prepared as KBr (Sigma Aldrich, Merck, Darmstadt, Germany) pellets (sample: KBr ratio of cca. 1:100). For each sample, 16 scans were performed at a resolution of 2 cm^−1^.

### 2.7. Determination of Aceclofenac Content by HPLC

Measurements were performed on a Thermo Scientific Finnigan Surveyor HPLC system, consisting of a quaternary pump, an autosampler with a temperature-controlled column compartment, and a diode array detector. A Thermo Scientific Betasil C18 (150 × 4.6 mm, 5 μm) chromatographic column was employed throughout the test, thermostatted at 55 °C. The mobile phase consisted of 0.1% (*v*/*v*) aqueous phosphoric acid solution: acetonitrile (45:55 *v*/*v*%), delivered with a 1.5 mL/min flow rate (retention time of aceclofenac was ~3.0 min). Quantitative determinations were performed at 270 nm, based on a five-point calibration curve (methanolic solution, at 10–100 μg/mL range). To determine the aceclofenac content, the microfibrous samples (n = 3) were dissolved in methanol and diluted 10-fold with the same solvent.

### 2.8. In Vitro Dissolution Studies

Small-volume, in vitro dissolution tests were performed as described in our earlier publication [41], in an in-house assembled dissolution apparatus. Dissolution tests were performed in a 25 mL dissolution medium (0.1 M phosphate buffer pH 6.8). The dissolution values were the average of three measurements. Aceclofenac (around 5–6 mg) and microfibrous samples (containing 5–6 mg aceclofenac, around 32–38 mg of fiber) were each placed into separate test tubes equipped with a 10 × 2 mm Teflon-coated magnetic stir bar. Stir rate was set to 200 rpm, using a magnetic stirrer, while the temperature of the dissolution medium was maintained at 37.0 °C ± 1 °C using an Erweka 1500i bath heat exchanger (Erweka GmbH, Heusenstamm, Germany). Samples of 1 mL were withdrawn at predefined time intervals (3, 5, 10, 15, 30, and 60 min), and filtered through a 0.45 μm RC syringe filter. Aceclofenac concentrations were determined using the same liquid chromatographic conditions as described above.

### 2.9. Residual Ethanol Content Determination of the Microfibrous Samples Using Gas Chromatography (GC)

The residual EtOH was determined by gas chromatography, performed on a Dani Master GC Gas Chromatograph with a flame ionization detector (FID) (Dani Instruments S.p.A, Milan, Italy), employing an Optima WAX capillary column (adsorbent thickness: 2 μm, diameter: 0.53 mm, length: 30 m), using the following temperature gradient program: 60 °C for 5 min, increase to 90 °C with a rate of 5 °C/minute; hold for 5 min. FID temperature was 250 °C, whereas the rest of the parameters were as follows: injector temperature: 250 °C, injection volume: 1 μL, carrier gas: nitrogen (10 mL/min), combustion gases: hydrogen (40 mL/min), synthetic air (220 mL/min), and nitrogen (25 mL/min).

### 2.10. Viscosity Measurement

The dynamic viscosity of the PVP solutions was measured with an IKA ROTAVISC lo-vi viscometer (IKA, Staufen, Germany). The measurements were performed using the SP3 spindle at 30 rpm at 21 °C. The viscosity values were recorded after 1 min of the measurement.

## 3. Results and Discussions

### 3.1. Fiber Mat Characterization

The production of aceclofenac-loaded PVP solid dispersions by electrospinning has been reported earlier [39]. In this study, we have kept PVP as the carrier polymer in order to be able to compare the dissolution results. However, the spinning conditions needed to be optimized for centrifugal spinning. We have carried out a systematic study to investigate the effect of rotational speed of the rotating chamber and PVP solution concertation on the morphology of the produced fiber mats and on the average fiber diameter.

**Figure 3:**
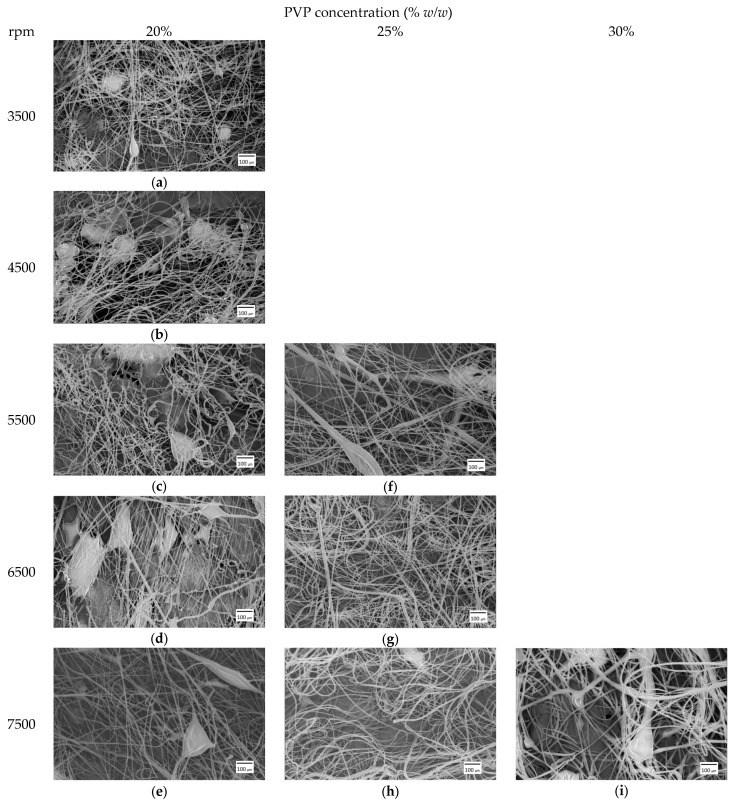
SEM images of PVP fiber mats spun at different rotation speed from 20% *w*/*w* (**a**–**e**), 25% *w*/*w* (**f**–**h**), and 30% *w*/*w* (**i**) PVP solution concentration at 100× magnification.

Figure 3 shows the SEM images of samples spun from 20, 25, and 30% *w*/*w* PVP solution. The fiber mats produced from 20% *w*/*w* PVP solution resulted in beaded fibers at all rotation speed values. There was no fiber production from the 25% *w*/*w* PVP solution at 3500 and 4500 rpm; however, beaded fibers appeared at 5500 rpm. Smooth-surfaced, bead-free, and randomly oriented fibers were produced at 6500 rpm, whereas at 7500 rpm a few beads started to appear again on the fiber mats. In the case of the 30% *w*/*w* PVP solution, fiber production started only at 7500 rpm rotational speed; however, these were beaded fibers. During centrifugal spinning, the surface tension has to be balanced with stress, resulting from pulling or tensile force acting on the fiber. The tensile force originates from the centrifugal force and the viscoelastic forces present during the process in the jet. It is known that at low concentrations (low viscoelastic forces), beaded fibers appear, and as the concentration increases, the beading disappears [42]. The dynamic viscosity of the prepared PVP solutions was measured to be 476, 1224, and 2460 mPa·s for 20, 25. and 30% *w*/*w* concentration, respectively. It can be observed that the concentration has a profound effect on the viscosity, and thus on the fiber production process. The viscosity of the prepared solutions increased ~2.5 times and ~5 times for 25 and 30% *w*/*w* concentration, respectively, when compared to the 20% *w*/*w* solution. From our results, it appears that the rotation speed also has a profound effect on the beading process, since bead-free fibers appeared at 25% *w*/*w* PVP concentration at a higher rotation speed. However, based on our observation, if the rotation speed further increases, beads and droplets start to appear again. This could be explained by the appearance of excess tensile forces in the jet resulting in a higher stress than the surface tension, and thus the jet breaks and beads form again.

Table 1 presents the tabulated average fiber diameters as a function of solution concentration and rotation speed. The results show that the rotation speed does not have a significant effect on the fiber diameter in our case. At 20% *w*/*w* concentration, the fiber diameters show little variation with speed and the standard deviation is also high. The increased standard deviation values could be due to the presence of higher-diameter outlier fibers that form in the beginning of the centrifugal spinning process [25]. As the solution concentration increases, the fiber diameter also increases, and thus at 7500 rpm, the fiber diameter increased from 4 ± 1.8 μm to 4.6 ± 1.6 μm and 6.8 ± 2.4 μm for 20, 25 and 30% *w*/*w,* respectively. PVP (K90) fiber production with centrifugal spinning has been reported earlier and for a 22% *w*/*w* PVP solution in 1:1 (v:v) EtOH:DMF solvent system 2.9 ± 1.1 μm fiber diameter was achieved at 6500 rpm [43]. The lower diameter compared to our results (4.3 ± 1.5 μm at 6500 rpm and 25% *w*/*w* concentration) could be explained by the evaporation rate difference of the solvent during the spinning process. Since a mixture of EtOH and DMF has a lower evaporation rate, due to the presence of the DMF, more time is provided for the jet to elongate and to reduce the diameter. In the case of pure EtOH, the evaporation rate is high, and thus the jet dries out very early in the process, resulting in higher diameter and a lower influence of rotation speed on the fiber diameter.

**Figure 4:**
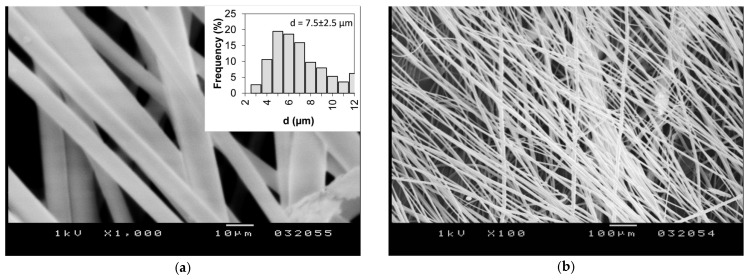
SEM images of the aceclofenac-loaded PVP fiber mat obtained by centrifugal spinning: (**a**) at 1000× magnification with an inset showing a histogram of the fiber size; (**b**) at 100× magnification.

Based on the morphology and fiber diameter results, the optimal centrifugal spinning conditions are achieved when 25% *w*/*w* PVP solution concentration is used at 6500 rpm, and thus this concentration and rotation speed was selected to prepare the drug-loaded fiber mat. Figure 4 shows sample SEM images of the obtained aceclofenac-loaded PVP fibers. It can be observed that the resulting fibers are smooth-surfaced and bead-free. The average fiber diameter was determined to be d = 7.5 ± 2.5 μm, which is ~1.7 times higher than the pure PVP fiber diameter spun using the same parameters. The production of aceclofenac-loaded PVP fibers lasted for 3 min and a total of 639 mg sample was collected. The production rate, normalized to 1 h can be calculated to be 12.7 g/h dry fibers. The productivity of aceclofenac-loaded PVP fibers by the electrospinning process was 0.07 g/h dry fiber [39], indicating that centrifugal spinning provides a production rate of two orders of magnitude higher than that of electrospinning. Production rate data was not reported in other papers dealing with the dissolution enhancement of aceclofenac [35,36,37,38].

### 3.2. Determination of Aceclofenac and Ethanol Content of the Microfibrous Webs

To confirm the presence of the active substance in the microfibrous formulation and to quantify its concentration, liquid chromatography measurements were employed. Based on the results of these measurements, the prepared webs contain 15.6 ± 0.2% (*w*/*w*) aceclofenac, which corresponds to 99 ± 1% of the theoretical (nominal) concentration (Table 2).

Since centrifugal spinning was performed under room temperature conditions from an ethanolic solution, it is important to track the residual solvent content of the produced fibers. According to current regulatory guidelines, ethanol is classified as a class 3 solvent (solvents with low toxic potential), with an upper limit of 0.5% [44]. Gas chromatographic determinations revealed that the ethanolic content of the dry fibrous mats was below the detection limit of the employed method (<0.10%), meaning that ethanol has almost completely evaporated during the fiber formation.

One drawback of the electrospinning process when it comes to the preparation of drug-loaded fibers is that one must take into consideration not only the viscosity and the surface tension but also the electric properties of the electrospinning solutions. In most cases, these properties depend not only on the characteristics of the fiber-forming polymers but also on the embedded drug substances and additives. Thus, there is a fine balance between the solvent used for preparing the electrospinning solution and the amount of drug that can be dissolved. As previously reported, the preparation of aceclofenac-containing nanofibrous mats from aqueous-ethanolic PVP solution needed an additive, triethanolamine, to facilitate the electrospinning process. Furthermore, low aceclofenac amounts could only be dissolved, 5.88% *w*/*w* compared to the PVP amount. This was mainly due to solubility constraints and the alteration of electric properties induced by the presence of the active substance in the polymeric solution.

In the case of centrifugal spinning, as the electric properties of the solution are not critical, a higher concentration of the active substance can be attained in the polymeric solution. Thus, in the present case, the aceclofenac content of the prepared fibrous formulation was around 2.7 times higher than in the case of the electrospun fibers (15.6% vs. 5.88% *w*/*w*).

### 3.3. Solid-State Characterization of the Obtained Microfibrous Webs

To track the physicochemical changes that intervene during the electrospinning process, solid-state characterization of the microfibrous webs was attained through DSC and FT-IR studies.

#### 3.3.1. DSC Study

**Figure 5:**
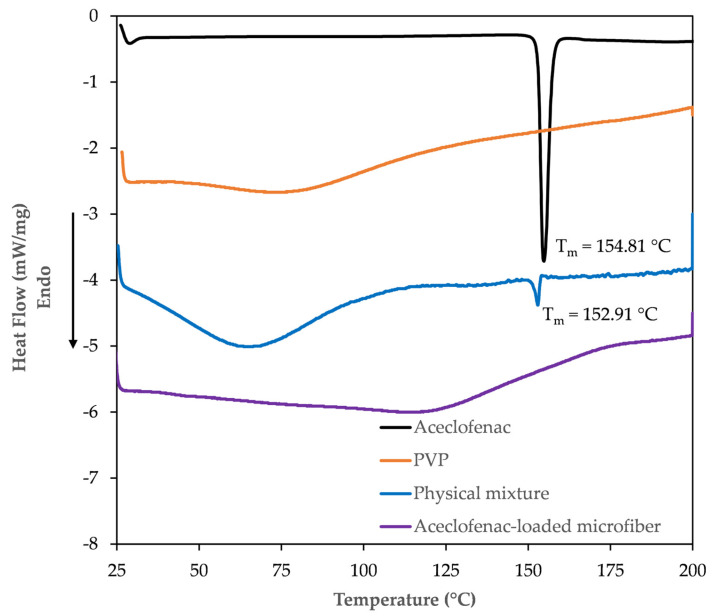
DSC thermograms of pure aceclofenac (black), PVP (orange), the physical mixture of aceclofenac and PVP (blue), and the aceclofenac-loaded PVP microfibers (purple).

Overlaid thermograms of the active substance, the fiber-forming polymer, their physical mixture, and the obtained fiber mats are presented in Figure 5. The thermogram of aceclofenac is characterized by the presence of a single sharp, endothermic peak at 154.81 °C, indicating the melting of the crystalline phase. The obtained values are in good correlation with the values described earlier in the literature [37]. The DSC thermogram of PVP K90 is characteristic of an amorphous, hygroscopic substance, with a broad endothermic event around 100 °C, indicating the dehydration of the polymer. In the thermogram of the physical mixture (prepared from the same amount of polymer and aceclofenac as in the case of the microfibers), the overlay of the thermograms of the two components can be observed. The endothermic peak of the active can clearly be observed in this thermogram at 152.91 °C, indicating its crystalline form. The melting point depression of aceclofenac in the physical mixture can be explained by possible interactions between the PVP and aceclofenac. The smaller endothermic peak intensity of the active refers to its proportion in the physical mixture. On the other hand, the DSC thermogram of the prepared microfibrous mats can only be characterized by a broad endothermic peak. The endothermic melting peak of the active substance completely disappears from the thermogram of the microfibers, indicative of the crystalline to amorphous transition of aceclofenac through fiber formation.

#### 3.3.2. FT-IR Study

**Figure 6:**
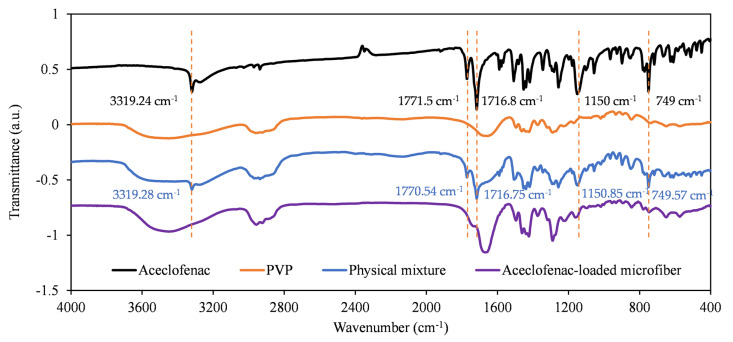
FT-IR spectra of pure aceclofenac (black), PVP (orange), physical mixture (blue), and the aceclofenac-loaded PVP microfibers (purple).

The FT-IR spectra of the individual components, their physical mixture, and the obtained fibrous mats are depicted in Figure 6. The individual spectra were shifted vertically to present them in one figure. The IR spectrum of aceclofenac is in accordance with earlier reports [39,45]. The characteristic peaks are in accordance with those described in the literature, with the most prominent one observed at the following wavenumbers: 3319.24 cm^−1^ (N-H stretching), carbonyl stretching at 1771.50 and 1714 cm^−1^, aromatic C=C stretching at 1589.49, 1508.19, and 1452.86 cm^−1^, and aromatic C-H stretching at 781 and 749 cm^−1^. The fiber-forming polymer, PVP, is characterized by a large band at 3450.59 cm^−1^ (O-H stretching), 2956 cm^−1^ (CH_2_ asymmetric stretching), 1655.33 cm^−1^ (C=O stretching), and some other peaks at 1495 cm^−1^ (C=N stretching), 1422.64, 1290 cm^−1^. As also observed in the DSC studies, the physical mixture can mostly be characterized by an overlay of the FT-IR spectra of the individual components. Although in some cases they are shifted, the characteristic peaks of aceclofenac can clearly be identified at 3319.28 (aceclofenac N-H stretch) and 1716.75 cm^−1^ (aceclofenac carbonyl stretch), but also at 1771.40, 1150.85, and 749.57 cm^−1^. Some of the mentioned peaks are highlighted in Figure 6 with orange dotted lines. These peaks almost completely disappear in the case of the FT-IR spectra recorded for the microfibrous formulation. The disappearance of these peaks and the general peak broadening observed in the FT-IR spectrum [45] of the microfibrous mats can further underline the hypothesis of the amorphous transition of the crystalline aceclofenac through fiber formation.

### 3.4. In Vitro Dissolution Studies

**Figure 7:**
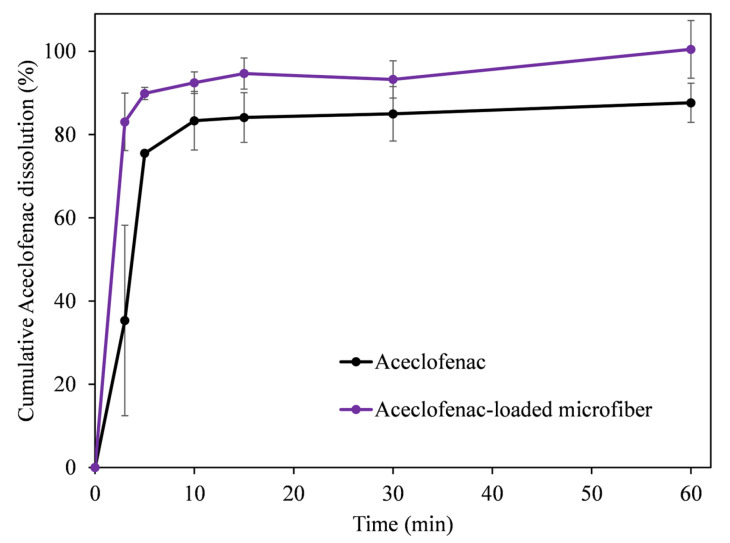
Dissolution profiles of pure aceclofenac and aceclofenac-loaded microfibers.

A small-volume dissolution study was undertaken using phosphate buffer pH 6.8. Figure 7 compares the in vitro drug release from the pure active pharmaceutical ingredient (API), aceclofenac, and the microfibrous formulation. The results indicate that the fiber-based formulation outperformed the active ingredient in all tested time points. More importantly, due to the rapid disintegration of the fiber mats, almost instantaneous release and improved solubility of the active can be seen. Larger differences were observed mostly at the initial time points, for example at 3 min only 35% was dissolved from the API, whereas in the case of the microfibers it was 83%. It can also be observed from the dissolution results, that after 60 min only ~87% of the aceclofenac was dissolved from the crystalline form, whereas ~100% was dissolved from the fibrous mat. The rapid release of aceclofenac from the fiber mats is most likely due to the large surface-to-volume ratio of the fibers, the amorphous state of the drug, and the hydrophilic nature of the chosen polymer.

Interestingly, the dissolution profile from the fiber mat prepared by centrifugal spinning is similar to the one obtained with the single nozzle electrospinning apparatus [39]. This indicates that, despite the 12 times larger fiber diameter and almost 2.7 times higher drug loading, the present formulation maintains its in vitro release characteristics and may be an interesting alternative for a fast-dissolving aceclofenac formulation. Furthermore, it also suggests that the binary system, drug and polymer carrier, is as effective as the ternary system that was used for electrospinning to increase the dissolution of aceclofenac and facilitate the electrospinning process.

Aceclofenac release data of chitosan-coated aceclofenac crystals showed slow release, and depending on the preparation conditions, the maximum cumulative release varied between 55–96% when using a dissolution medium of 0.1N HCl containing 2% Tween 80. It should be noted that the release rate was slow compared to our results, with ~80% being the highest after 30 min [35]. Rameshwar et al. used polymeric microspheres to enhance the dissolution characteristics of aceclofenac. The drug loading varied between 20–28% during their experiments with a maximum encapsulation efficiency of 84%. The best dissolution result was ~80% after 13 h. However, the maximum dissolution was ~35% after 30 min in phosphate buffer [36]. Pattnaik et al. prepared aceclofenac nanocrystals to enhance the dissolution characteristics. The dissolution experiments were carried out in 1% sodium lauryl sulfate (SLS) solution in distilled water and the maximum relative dissolution was ~85% after 60 min, whereas it was ~80% after 30 min [37]. Jana et al. prepared chitosan-locust bean gum, interpenetrating networks to enhance the dissolution of aceclofenac in a slow and sustained fashion. The drug loading was kept at 25% compared to the carrier system. The maximum drug encapsulation was ~80%, whereas the maximum release reached ~95% after 8 h in phosphate buffer solution (pH 6.8) [38].

Based on the comparison of our results to the literature, one can conclude that fiber-based solid dispersions are an important contender to improving the solubility of aceclofenac. The drug loading is comparable, 15.6% compared to ~20% [38] and ~23% [36], respectively, after accounting for drug encapsulation efficiency. Furthermore, the preparation of fiber-based aceclofenac-containing solid dispersion is a one-step process (in contrast to the abovementioned processes) that provides ease and simplicity with excellent encapsulation efficiency.

## 4. Conclusions

In conclusion, aceclofenac-loaded PVP microfiber solid dispersion was successfully prepared by the centrifugal spinning method. The centrifugal spinning process was optimized to produce PVP fibers using EtOH as solvent. We found that not only the solution concentration but also the rotation speed have a profound effect on the fiber morphology. Bead-free fibers were obtained at 25% *w*/*w* PVP concentration and at 6500 rpm resulting in a fiber diameter of d = 4.3 ± 1.5 μm. Using the optimized conditions, smooth-surfaced and bead-free aceclofenac-loaded PVP fibers were produced with a fiber diameter of 7.5 ± 2.5 μm. The productivity of the centrifugal spinning process was calculated to be 12.7 g/h, two orders of magnitude higher than electrospinning. Fiber-based solid dispersion preparation by centrifugal spinning did not require the use of any additive. Furthermore, due to the minimal effect of the electrical conductivity of the used polymer-drug solution on the centrifugal process, the drug-loading of the fibers could be increased by 2.7 times with respect to electrospinning. The DSC and FTIR measurements confirmed the crystalline to amorphous transition of the active substance during the centrifugal spinning process. The dissolution study revealed a higher dissolution rate from the microfiber solid dispersion compared to the pure drug during the test. It was also found that the dissolution of aceclofenac from the solid dispersion prepared by centrifugal spinning is similar to the electrospun formulation, despite the higher fiber diameter. Furthermore, the dissolution result of the prepared fiber-based solid dispersion is on par with or surpasses te performance of other aceclofenac dissolution-enhancing formulations. The above-mentioned results lay the foundation for centrifugal spinning and prove that it is as easily usable and reliable method as electrospinning with the added benefit of high production rate and more flexible polymer–drug–solvent formulation when it comes to the preparation of drug-loaded fiber-based solid dispersion.

## Figures and Tables

**Table 1:** Tabulated average fiber diameter values.

d (μm)						
Concentration (*w*/*w*)						
Rpm	20%	Beads	25%	Beads	30%	Beads
3500	3.6 ± 1.1	Yes	--	--	--	--
4500	3.7 ± 1.1	Yes	--	--	--	--
5500	4.8 ± 1.8	Yes	3.8 ± 2	Yes	--	--
6500	3.2 ± 1.6	Yes	4.3 ± 1.5	No	--	--
7500	4 ± 1.8	Yes	4.6 ± 1.6	Yes	6.8 ± 2.4	Yes

**Table 2:** Aceclofenac content of the prepared microfibers according to HPLC measurements.

	Concentration (*w*/*w*%)			Concentration (% of Theoretical Concentration)		
Nr.	1.	2.	3.	1.	2.	3.
Value	15.49	15.83	15.59	98.40	100.58	99.06
Avg. ± st. dev.	15.6 ± 0.2			99 ± 1		

## Data Availability

The data presented in this study are available on request from the corresponding author.
